# A New Brain Drug Delivery Strategy: Focused Ultrasound-Enhanced Intranasal Drug Delivery

**DOI:** 10.1371/journal.pone.0108880

**Published:** 2014-10-03

**Authors:** Hong Chen, Cherry C. Chen, Camilo Acosta, Shih-Ying Wu, Tao Sun, Elisa E. Konofagou

**Affiliations:** 1 Department of Biomedical Engineering, Columbia University, New York, New York, United States of America; 2 Department of Radiology, Columbia University, New York, New York, United States of America; Hungarian Academy of Sciences, Hungary

## Abstract

Central nervous system (CNS) diseases are difficult to treat because of the blood-brain barrier (BBB), which prevents most drugs from entering into the brain. Intranasal (IN) administration is a promising approach for drug delivery to the brain, bypassing the BBB; however, its application has been restricted to particularly potent substances and it does not offer localized delivery to specific brain sites. Focused ultrasound (FUS) in combination with microbubbles can deliver drugs to the brain at targeted locations. The present study proposed to combine these two different platform techniques (FUS+IN) for enhancing the delivery efficiency of intranasally administered drugs at a targeted location. After IN administration of 40 kDa fluorescently-labeled dextran as the model drug, FUS targeted at one region within the caudate putamen of mouse brains was applied in the presence of systemically administered microbubbles. To compare with the conventional FUS technique, in which intravenous (IV) drug injection is employed, FUS was also applied after IV injection of the same amount of dextran in another group of mice. Dextran delivery outcomes were evaluated using fluorescence imaging of brain slices. The results showed that FUS+IN enhanced drug delivery within the targeted region compared with that achieved by IN only. Despite the fact that the IN route has limited drug absorption across the nasal mucosa, the delivery efficiency of FUS+IN was not significantly different from that of FUS+IV. As a new drug delivery platform, the FUS+IN technique is potentially useful for treating CNS diseases.

## Introduction

Central nervous system (CNS) diseases are difficult to treat as most systemically administered therapeutic agents cannot penetrate the blood-brain barrier (BBB). The BBB prevents the access of ∼100% of large-molecule drugs and ∼98% of small-molecule drugs (usually defined as smaller than around 500–600 Da in molecular weight [1]) to the brain from the systemic circulation [2]. Various strategies have been developed for circumventing the BBB, such as invasive direct injection or infusion, modification of therapeutic agents, and carrier-mediated transport [3]. Among these strategies, intranasal (IN) delivery is a non-invasive approach for direct drug delivery to the brain via the nose, bypassing the BBB [4].

IN administration has emerged as a promising approach for drug delivery to the brain. Traditionally, it has been successfully used as a convenient method for drug delivery to the systemic circulation, because IN administered drugs can be absorbed through a rich vascular network in the nasal cavity into the systemic circulation [5]. Although most drugs absorbed into the blood circulation cannot enter the brain parenchyma because of the BBB, it has been demonstrated feasible in animal and clinical studies that drugs can be directly delivered from the nostrils to the brain, bypassing the BBB. A wide-range of therapeutics, such as peptides, proteins, gene vectors and stem cells, have been successfully delivered through IN administration to small animal brains and have shown efficacy in treating CNS diseases, such as Alzheimer's disease, Parkinson's disease, Huntington's disease, depression, anxiety, autism spectrum disorders, seizures, drug addiction, eating disorders, and stroke [6]–[8]. Moreover, a number of clinical trials have demonstrated CNS effects in humans following IN delivery of biologics, among which IN administration of insulin has been found to be a promising approach to slow down the progression of Alzheimer's disease [9].

Despite the fact that the mechanisms involved in IN drug delivery to the brain are still being elucidated, some of the pathways involved are known. The neural connections between the nasal mucosa and the brain through the olfactory and trigeminal nerves (involved in sensing odors and chemicals) have been found to provide unique pathways for the noninvasive delivery of therapeutic agents to the CNS [4], [6]. IN administered drugs are most likely transported extracellularly along the olfactory and trigeminal nerves to the brain. The distribution of drugs from these brain entry points to other distant CNS sites may be envisioned to occur either by intracellular or extracelluar transport. The extracellular transport can occur via convective transport within the cerebral perivascular spaces, local diffusion at the entry points, and local diffusion from perivascular spaces into the parenchyma [4], [6]. Although promising in brain drug delivery, the IN route has some disadvantages. One major drawback of this route is that the fraction of drug reaching the CNS from the nasal cavity is small, which has restricted its application to very potent substances [6], [10]. The other disadvantage is that drugs are delivered to the whole brain through this route, while neurological diseases do not generally affect the brain in a global manner. Therefore, new strategies are needed for enhancing drug delivery efficiency at the sites requiring treatment while minimizing the exposure to other brain sites. Transcranial focused ultrasound (FUS) in combination with microbubbles may offer such a strategy.

FUS in combination with microbubbles can increase the BBB permeability for targeted brain drug delivery with no or minimal tissue damage [11], [12]. The experimental design typically uses intravenous (IV) injection of therapeutic agents with microbubbles, and then applies FUS to induce BBB opening for drug delivery. The FUS focuses externally generated ultrasound pulses through the skull onto a small focal region (on the order of millimeters) deep into the subcortical structures, which allows highly precise and noninvasive targeting of brain regions where treatment is desired. Microbubbles are micron-scale gas bubbles stabilized by a lipid, protein, albumin or polymer shell. They have been used as ultrasound imaging contrast agents in the clinic for more than two decades and have shown great potential as drug delivery agents for therapeutic applications. The FUS technique has been successfully used in the delivery of various therapeutic agents, such as chemotherapeutic drugs [13], neurotrophins [14], antibodies [15], gene vectors [16], and cells [17]. Our group and others have shown that the BBB opening induced by FUS is reversible, and its closing time ranges from several hours to several days, depending on the acoustic parameters [18]. The volume of BBB opening can be controlled by adjusting the acoustic parameters or microbubble sizes [19]. Previous short-term (up to 5-hr survival) and long-term (up to 4-wk survival) safety assessment of the FUS technique demonstrated that, when appropriate ultrasound parameters are used, there is no or only minimal histological damage [20], [21]. Nevertheless, although IV injection of therapeutic agents has high bioavailability, it is associated with systemic exposure and thus not suitable for the delivery of drugs with short half-lives in blood and/or high systemic side effects, such as neurotrophic factors, neuropeptides, and hormones [8], [22]. In contrast, IN administration is a painless, simple, and noninvasive approach that allows direct drug delivery to the brain, eliminating the need for systemic delivery and thereby reducing associated side effects [7], [8].

The physical mechanisms for FUS-enhanced drug delivery have been under extensive investigation [23], [24]. Different from other contrast agents used in medical imaging, microbubbles are confined within the blood vessels after IV injection due to their relatively large sizes. When microbubbles pass through the FUS focal region, they cavitate, which is a broad term for ultrasound-induced activities of bubbles, including their oscillation and collapse. Cavitation is usually divided into two classes: stable cavitation (bubbles stably oscillate) and inertial cavitation (bubbles rapidly collapse). The cavitation emissions from microbubbles during FUS sonication can be detected, allowing real-time monitoring of the FUS treatment [25]. Microbubble cavitation generates mechanical effects on the nearby blood vessels, such as high shear stress, microstreaming, and microjetting, enabling transvascular delivery of drugs in the blood circulation [26]. Meanwhile, the oscillating microbubbles can push and pull on the blood vessels along with surrounding tissues, inducing expansion and contraction of the perivascular spaces [27]. The displacements of the perivascular spaces may induce convective bulk fluid flow, leading to enhanced drug penetration. Moreover, the radiation force generated by the FUS beam itself without microbubbles can generate shear stress on the tissue and increase hydraulic conductivity of the interstitial space, which can increase drug diffusion [26]. The mechanical effects exerted by the FUS beam in combination with microbubbles contribute to the enhanced brain delivery of IV-injected therapeutic agents. These same mechanical effects may also enhance the delivery of drugs administered through the IN route.

Therefore, we hypothesized that FUS can enhance IN drug delivery efficiency at the targeted brain sites. There were two objectives of this study: (1) to explore the feasibility of this new brain drug delivery strategy–FUS in combination with IN (FUS+IN); (2) to compare the drug delivery outcomes of this new strategy with the conventional approach–FUS coupled with IV injection (FUS+IV). Wild-type mice were used as the animal model and a 40 kDa fluorescently-labeled dextran was used as the model drug [28]. Dextrans are glucose polymers and the 40 kDa dextran was selected as the model drug because its molecular weight is on the same order of magnitude as many neurotrophic factors that have considerable potential in the treatment of CNS diseases [22]. The drug delivery outcomes using different strategies were evaluated based on fluorescence images of brain slices.

## Materials and Methods

### Animal preparation

This study was carried out in strict accordance with the recommendations in the Guide for the Care and Use of Laboratory Animals of the National Institutes of Health. The protocol was approved by the Columbia University Institutional Animal Care and Use Committee. All surgery was performed under isoflurane anesthesia, and all efforts were made to minimize animal suffering. The animal body temperature was maintained using a heating pad.

A total of 26 male C57BL/6 mice (20–25 g in weight; Harlan Laboratories, Indianapolis, IN) were used in this study. Among these 26 mice, 20 were divided into the following four experimental groups with n = 5 for each group. (1) Control group: no dextran delivery and no FUS. (2) IN sham group: IN administration of the dextran without FUS. (3) IN treatment group: IN administration of the dextran with FUS applied on the left side of the caudate putamen while the contralateral right side was not sonicated. (4) IV treatment group: IV injection of the dextran with FUS applied on the left side of the caudate putamen while the contralateral right side was not sonicated. The remaining 6 mice were treated following the same protocol as groups 3 and 4 with n = 3 for each group for the purpose of assessing the safety of the FUS treatment.

### Microbubble generation

Microbubbles comprised of a 90 mol% 1,2-distearoyl-sn-glycero-3- phosphocholine (DSPC) and 10 mol% 1,2-distearoyl-sn-glycero-3- phosphoethanolamine-N-[methoxy(polyethylene glycol)2000] (DSPE-PEG2000) (Avanti Polar Lipids, Alabaster, AL, USA) lipid-shell and a perfluorobutane (FluoroMed, Round Rock, TX, USA) gas-core were manufactured in-house. Size-selected microbubbles with a median diameter of 4–5 µm were isolated from a poly-dispersed microbubble distribution using a differential centrifugation method [29]. Their size distributions and concentrations were determined by a particle counter (Multisizer III, Beckman Coulter Inc., Opa Locka, FL, USA). Before each injection into the mouse, their concentrations were diluted using sterile saline to a final concentration of approximately 8×10^8^ number of microbubbles per mL.

### Administration of the dextran

In the control group (group 1), no dextran was administered.

In the IN sham group (group 2) and IN treatment group (group 3), ∼2 mg of 40 kDa Texas Red-labeled dextran (Life Technologies Inc, Grand Island, NY, USA) was administered intranasally following procedures used before [4], [30]. The dextran was dissolved in saline at a concentration of 40 mg/mL. The anaesthetized mice were placed supine with the head position stabilized horizontally. A micropipette was used to intranasally administer 3 µL drops of the dextran solution to alternating nostril every 2 minutes. Drops were placed at the opening of the nostril, allowing the animal to snort each drop into the nasal cavity. A total of 51 µL dextran solution (∼2 mg dextran) was delivered over a course of 34 min.

For the IV treatment group (group 4), the same amount of dextran (51 µL in volume, 40 mg/mL in concentration, and ∼2 mg in dose) was administered by IV bolus injection through the tail vein.

### Focused ultrasound sonication

**Figure 1:**
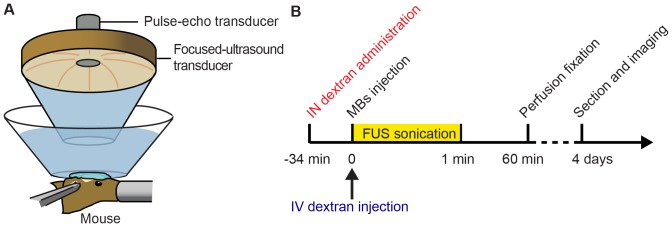
Illustration of experimental (A) setup and (B) timeline. For IN administration, the dextran was administered 3 µL at a time, alternating between the two nostrils, with a lapse of 2 min between each administration, over a total of 34 min.

For the IN treatment group (group 3) and IV treatment group (group 4), the mice were sonicated at a targeted brain location using an experimental setup illustrated in Fig. 1(A) and following an experimental timeline shown in Fig. 1(B).

A single-element FUS transducer (center frequency: 1.5 MHz, focal depth: 60 mm, diameter: 60 mm; Imasonic, Besancon, France) was driven by a function generator (33220A, Agilent, Palo Alto, CA, USA) through a nominal 50 dB gain power amplifier (325LA, E&I, Rochester, NY, USA). The lateral and axial dimensions of the FUS focal region measured in water were 1.2 mm and 13.0 mm. A custom-built truncated cone was attached to the transducer and filled with degassed water to provide acoustic coupling. The cone was immersed in a degassed-water container. The bottom of the water container had a window sealed with an almost acoustically and optically transparent membrane. The container was placed on the mouse head and coupled with degassed ultrasound gel. Acoustic emissions arising from microbubble cavitation were acquired by a pulse-echo transducer (center frequency 10 MHz; focal length 60 mm; Olympus NDT, Waltham, MA, USA). It was positioned through a central hole of the FUS transducer and confocally aligned with the FUS transducer. The signals received by the pulse-echo transducer were amplified by 20 dB (Model 5800, Panametrics-NDT, Waltham, MA, USA) and then digitized (Razor Express CompuScope 1422, Gage Applied Technologies, Inc., Lachine, QC, Canada) at a sampling frequency of 50 MHz.

Before FUS sonication, each mouse was positioned prone with its head immobilized by a stereotaxic frame (David Kopf Instruments, Tujunga, CA, USA). The fur on the mouse head was removed with an electric clipper and a depilatory cream. A modified 27G×½ butterfly catheter (Terumo Medical, Somerset, NJ, USA) was inserted into the tail vein for IV injection. The FUS transducer was moved 2 mm lateral of the sagittal suture and 6 mm anterior of the lambdoid suture using a previously described grid positioning method [31]. Freshly diluted microbubble suspension (30 µL) was administered through a bolus injection via the tail vein prior to each sonication. For the IV treatment group (group 4), the microbubbles were co-injected with the dextran (Fig. 1B). Immediately after injection (∼5 s), pulsed FUS (center frequency: 1.5 MHz; peak-negative pressure: 0.45 MPa; pulse length: 6.7 ms; pulse repetition frequency: 5 Hz; duration: 1 min) was applied transcranially to the left caudate putamen. The non-sonicated right caudate putamen served as control for IN administration only (group 3) or IV injection only (group 4). Additionally, prior to microbubble injection, a 30-s sonication using the same acoustic parameters was applied in order to measure the background cavitation signals, needed in the acoustic emission analysis described later.

For all the mice used in the current study, a 1-h period was allowed after finishing IN and IV dextran administration to enable the dextran to diffuse into the brain parenchyma (Fig. 1B). At the end of the allotted time, all mice were sacrificed by transcardial perfusion. The mouse brains were processed and prepared for either frozen (60 µm thick) or paraffin (6 µm thick) sections. The frozen sections were imaged by a fluorescence microscope (BX61; Olympus, Melville, NY, USA) and used later for quantifying dextran delivery outcomes. The paraffin sections were used for whole brain histological examinations by hematoxylin and eosin (H&E) staining [21].

### Acoustic emission analysis

The acoustic emission analysis method was the same as that described previously [32]–[34]. To quantify the stable and inertial cavitation behaviors of the microbubbles within the FUS targeted region, the stable cavitation dose and inertial cavitation dose were calculated, respectively.

For each ultrasound pulse, its frequency spectrum between 3 and 9 MHz was used for stable and inertial cavitation quantification to eliminate any contributions from the FUS beam (frequency  = 1.5 MHz). The broadband emission from inertial cavitation was obtained using a comb filter, which had a cut-off band around each harmonic and ultraharmonic frequencies with bandwidths of 350 kHz and 100 kHz, respectively. The root mean square of the filtered spectra was calculated to represent the inertial cavitation level for each pulse. The stable cavitation level was obtained by first calculating the root mean square of the amplitudes of all harmonics and then subtracting the corresponding inertial cavitation level. The inertial cavitation activity was thus not included in the stable cavitation quantification. The stable and inertial cavitation doses were calculated by integrating the stable and inertial cavitation levels over the total ultrasound exposure time, respectively. The final doses were then calculated by subtracting baseline doses obtained based on signals acquired prior to microbubble injection.

### Fluorescence analysis

The dextran delivery outcomes were determined by quantifying the fluorescence intensities within the targeted caudate putamen. Nine horizontal sections with four dorsal sections, four ventral sections, and a reference midline section were selected from each brain for the analysis. All the fluorescence images were first normalized by their corresponding exposure time. Then, a circular region-of-interest (ROI, diameter  = 1.2 mm) was manually aligned with the sonicated and control caudate putamen on each section, and the spatial average fluorescence intensity within the ROI was calculated using ImageJ (National Institutes of Health; Bethesda, MD). The diameter of the ROI was selected to be the same as the FUS transducer lateral focal region dimension (1.2 mm). The reported fluorescence intensity for each side of the brain was the sum of the calculated fluorescence intensities within the ROI of all nine sections.

### Statistical analysis

An unpaired two-tailed Student's t-test using GraphPad Prism (Version 5.01, La Jolla, CA, USA) was used to compare between groups. A P value of 0.05 was considered to represent a significant difference in all the analyses. All data were expressed as mean ± standard deviation.

## Results

### IN delivery to the brain

**Figure 2:**
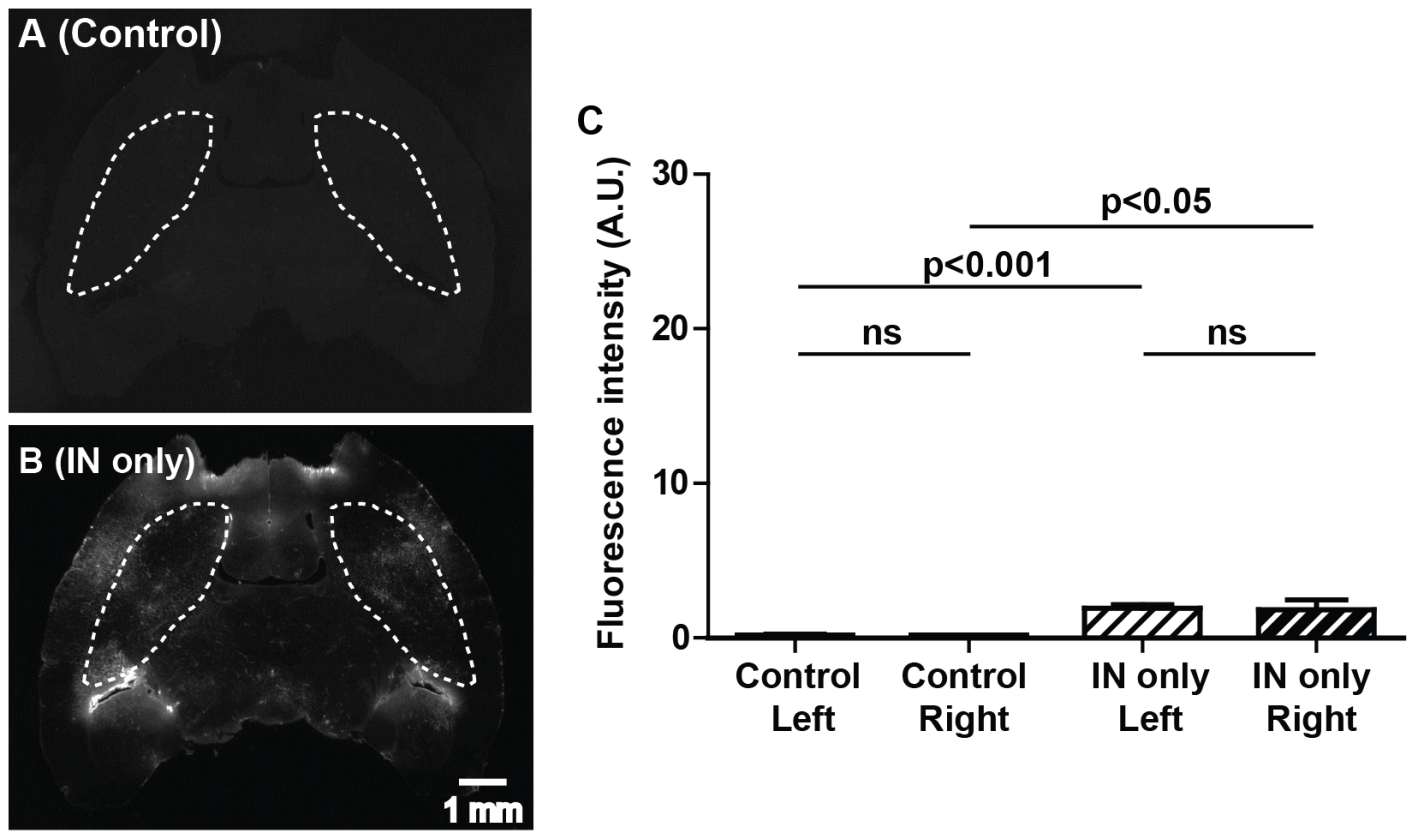
Fluorescently-labeled dextrans delivered to the whole brain through IN administration. (A) and (B) show the representative fluorescence images of horizontal sections of the whole brain from the control group (no FUS and no dextran administration) and the IN sham group (IN administration without FUS), respectively. The caudate putamen regions are highlighted by dash lines. (C) Quantitative analysis of the fluorescence intensities within the left and right caudate putamen regions for these two groups. IN administration resulted in a significant increase in the fluorescent intensity compared with the control. Scale bar represents 1 mm.

Figures 2A and 2B present representative fluorescence images of horizontal sections of the whole brain from the control group (group 1) and IN sham group (group 2). As shown in Fig. 2B, IN administration of the dextran without FUS resulted in an elevation of dextran concentration in the whole brain. A statistically significant increase in the fluorescence intensity was found in the IN sham group when compared with the control group (Fig. 2C), suggesting that the IN route enables direct access of drugs to the brain. Within each group, no difference was found between left and right caudate putamen regions, as expected. However, the delivered dextran did not accumulate in any particular brain region and the concentration achievable in different regions of the brain varied, which confirmed the non-targeted nature of IN administration.

### FUS-enhanced IN delivery

**Figure 3:**
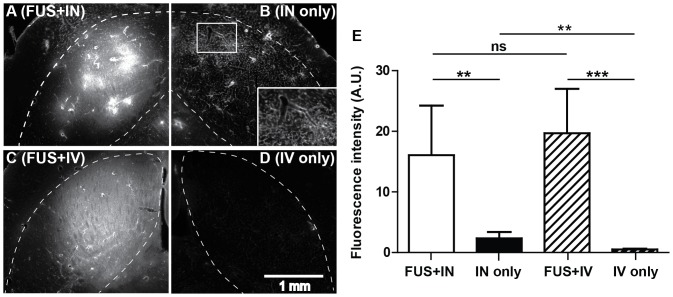
Focused ultrasound enhanced targeted delivery of the dextran administered through IN or IV route. (A) and (B) show fluorescence images of part of the left and right caudate putamen in one horizontal section from one mouse in the IN treatment group. (C) and (D) show part of the left and right caudate putamen in one horizontal section from one mouse in the IV treatment group. FUS was targeted at the left caudate putamen. The caudate putamen region in each image was highlighted by the dash line. The box insert in (B) is a blow up of the square area in (B), which shows that the vascular network was highlighted by the dextran, confirming that IN administered compounds can be transported within the perivascular spaces of cerebral blood vessels. (E) Quantitative fluorescence intensity analysis within the left and right caudate putamen regions for these two groups. FUS significantly enhanced IN delivery efficiency. No difference was found between FUS+IN and FUS+IV. Scale bar represents 1 mm.

Figures 3A and 3B show that FUS exposure in the presence of microbubbles significantly enhanced IN delivery at the targeted left caudate putamen (Fig. 3A) when compared with the contralateral right side with IN administration only (Fig. 3B). Quantification of the fluorescence intensities of mouse brains in the FUS+IN group found an 8-fold increase in the fluorescence intensity compared with the contralateral control side with IN only (Fig. 3E).

When IV injection was used for dextran delivery instead of IN, localized dextran accumulation was observed at the targeted caudate putamen. Figures 3C and 3D display representative fluorescence images from the FUS+IV treated (left) caudate putamen and contralateral non-treated (right) side with IV dextran injection only. The two mouse brains shown in Figs. 3A, B and 3C, D had fluorescence intensities of similar magnitude (27.2 and 19.4, respectively). When comparing across the entire FUS+IN and FUS+IV groups, the administration route did not appear to affect the delivery efficiency in the targeted caudate putamen as no significant difference in dextran accumulation was detected between FUS+IN and FUS+IV groups (P = 0.48; Fig. 3E). In addition, a significant increase in fluorescence intensity was found in the IN only group (Fig. 3B) compared with IV only group (Fig. 3D) (P = 0.005; Fig. 3E), further confirming that IN administration alone allows the dextran to gain direct access to the brain.

It should be noted that although the fluorescence intensities of FUS+IN and FUS+IV groups were at a similar level, distinct dextran distribution characteristics were observed, as representatively shown in Figs. 3A and 3C. Images shown in Fig. 3A–D were acquired at higher magnification than those presented in Fig. 2 to highlight the features of dextran distribution. A single sonication following IV dextran injection resulted in a more homogeneous dextran distribution within the FUS targeted region (Fig. 3C), while a less homogeneous dextran distribution with high intensity regions was observed in the FUS+IN group (Fig. 3A). Heterogeneous dextran distribution was also observed on the contralateral control side with IN administration only as shown in Fig. 3B as well as Fig. 2B. In addition, it was clearly observed in Fig. 3B that the vascular network was highlighted by the dextran, confirming that IN administered compounds can be transported within the perivascular spaces of cerebral blood vessels [7].

**Figure 4:**
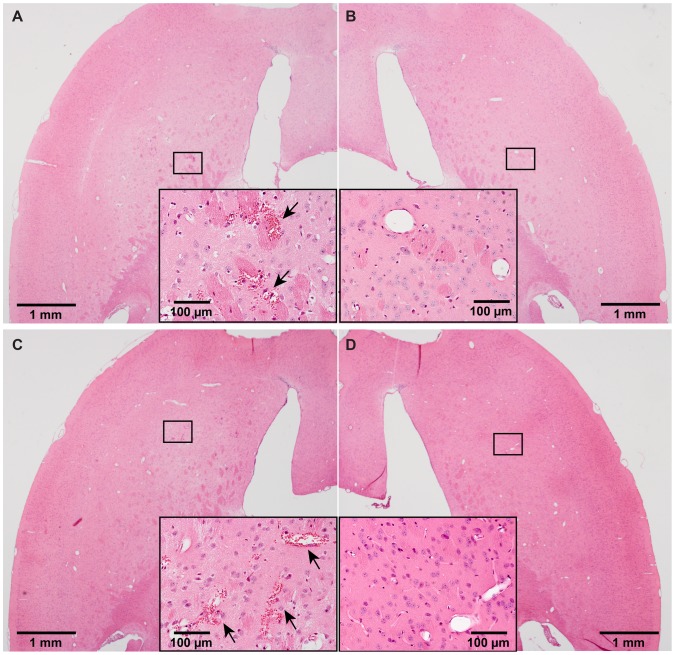
Whole brain histological examinations by hematoxylin and eosin (H&E) staining. Microscopic examination of (A, C) left (sonicated) and (B, D) the corresponding right (nonsonicated) caudate putamen in H&E stained horizontal sections. (A) and (B) were from the same section of a mouse brain with the most severe damage in the FUS+IN group. (C) and (D) were from the same section of a mouse brain with the most severe damage in the FUS+IV group. Minor microhemorrhages, as pointed out by arrows, were observed on the FUS targeted left caudate putamen in (A) and (C). No tissue damage was observed on the contralateral right caudate putamen without FUS sonication in (B) and (D).

**Figure 5:**
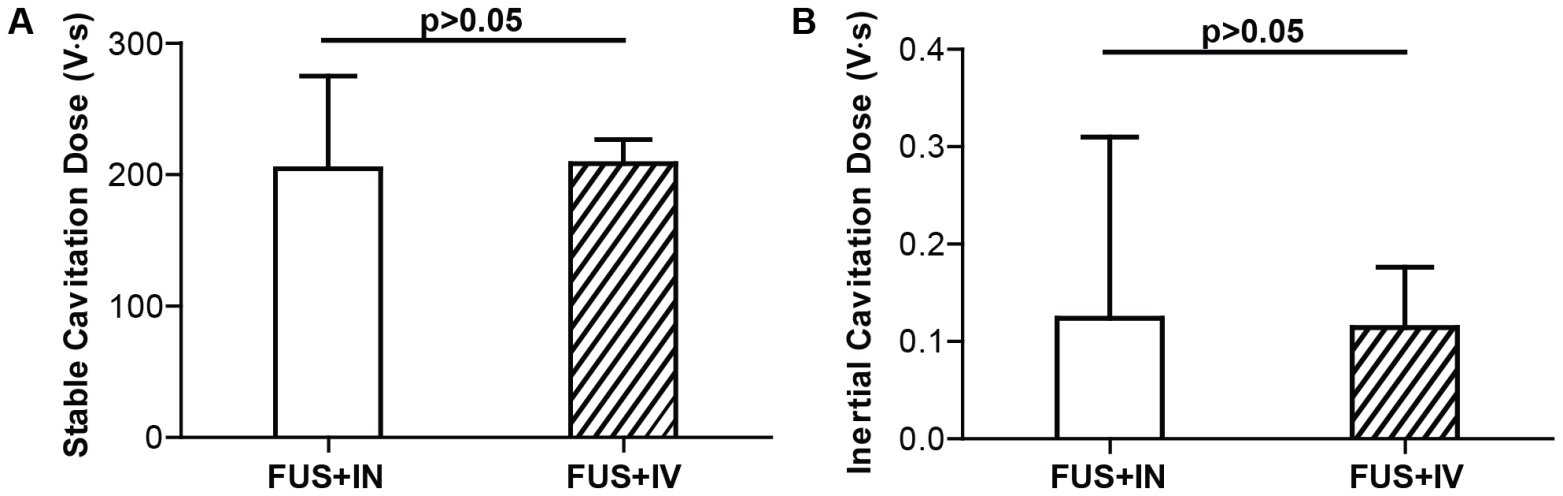
Quantification of microbubble cavitation activities in FUS+IN and FUS+IV groups. (A) Stable cavitation doses. (B) Inertial cavitation doses. No difference was found in both stable cavitation behavior and inertial cavitation behavior between these two groups.

Preliminary safety assessment of FUS sonication was performed by histological analysis. Minor microhemorrhages were observed in both FUS+IN and FUS+IV groups. Figures 4C and 4D depict cases with the most severe damage from the FUS+IN and FUS+IV groups, respectively. Small clusters of erythrocyte extravasation were observed in both cases. Figure 5 shows the microbubble activities detected in these two groups, as quantified by stable and inertial cavitation doses. No significant difference in stable or inertial cavitation dose was found between the FUS+IN and FUS+IV groups.

## Discussion

Our findings validated the hypothesis that FUS can enhance the delivery of IN-administered drugs at a targeted brain location. It is the first time that this novel combination of these two different platform techniques is proposed. Numerous studies have demonstrated that the IN route can provide a non-invasive approach for brain drug delivery, but none of them have shown that an enhanced delivery at a targeted location was possible. The FUS+IN technique offers a potentially useful drug delivery platform for treating CNS diseases.

By combining FUS with IN drug delivery, we achieved a significant increase in drug accumulation in the targeted brain site, thus improving the bioavailability and biodistribution of the IN administered drug. We confirmed that IN-administered dextran could be delivered to the brain, bypassing the BBB, but it had low delivery efficiency and was non-targeted (Fig. 2). IN administration in combination with FUS achieved an 8-fold increase in drug delivery efficiency within the targeted region compared with IN administration only (Fig. 3E). In the past, the IN route could only be used for particularly potent substances because of limited absorption across the nasal epithelium [6]. FUS+IN may expand its application to a much broader range of drugs. Moreover, although IN administrated drugs are distributed to the whole brain, by decreasing the IN administration dose, FUS+IN has the potential to achieve therapeutic drug level only within the targeted site while keeping non-targeted sites at sub-therapeutic level. Therefore, the FUS technique would potentially overcome the major disadvantages of IN brain drug delivery: (1) overcome the restriction that the IN route can only be used for particularly potent substances and (2) achieve targeted brain drug delivery. FUS+IN can be applied in the future for the delivery of drugs that have been previously shelved from IN administration due to their poor pharmacokinetics. New CNS therapeutic strategies could emerge based on the FUS+IN technique.

Our findings should not be interpreted as if to establish that the IN administration when combined with FUS is superior for targeted brain drug delivery than FUS+IV. On the contrary, FUS+IV is better at targeted delivery than FUS+IN. IN administration is associated with drug delivery to the whole brain, whereas the dextran injected through IV can only be delivered at the FUS targeted region where the BBB was opened. Therefore, for drugs exhibiting high stability in circulation and low systemic side-effects, such as dextrans (the half-life of the 40 kDa dextran in blood is about 7.5 hours [35]) used in this study, FUS+IV remains an effective technique since it provides drug access only to the targeted location without affecting other CNS sites. However, IN administration offers a non-invasive alternative over the more conventional IV injection for brain drug delivery. It allows direct access to the CNS and has low systemic bioavailability [22]. Therefore, FUS+IN may become a viable alternative for FUS+IV in the delivery of therapeutics that have beneficial effects within the CNS, but short half-lives in the blood and/or high systemic side effects. IN delivery of such therapeutics, such as neurotrophic factors, neuropeptides, and hormones, have been shown promise in the treatment of CNS diseases and injuries, including Alzheimer's disease, stroke, Parkinson's disease, motor neuron diseases, demyelinating diseases and traumatic brain injury [6], [8], [22]. The combination of IN administration and FUS has great potential to improve treatment outcomes of these diseases compared with IN administration alone.

We clearly showed brain uptake of the IN administrated dextran in both the IN only group and FUS+IN group (Figs. 2B, 3A, and 3B). In addition, we found the FUS+IN group showed less homogeneous dextran distribution (Fig. 3A) than the FUS+IV group (Fig. 3C). Similar heterogeneous dextran distribution was also observed in the IN only group (Figs. 2B and 3B). For drug delivery by FUS+IV, the drug circulated throughout the body and significant CNS delivery can only be achieved in brain locations where BBB opening is induced by FUS with microbubbles through cellular mechanisms, including opening a part of the tight junctions, formation of channel and fenestration in endothelial cell cytoplasm, and enhancing transcytosis [36]. On the other hand, IN administered drugs are most likely transported along the olfactory and trigeminal nerves to the brain entry sites and then distribute to other distant CNS sites via convective and diffusive transport from the cerebral perivascular spaces into the parenchyma [4], [6]. Figure 3B confirmed that IN-administered drugs can be transported within the perivascular spaces of cerebral blood vessels. The distribution of IN-administered drugs in the brain will be affected by the structural characteristics of the different brain compartments and the functional barriers between them [22], which may contribute to the observed heterogeneous dextran distribution. Future investigation is needed to better understand the pharmacokinetics of drug delivery by FUS+IN.

Further investigation is warranted on the mechanisms for FUS-enhanced IN drug delivery. It has been demonstrated that FUS and microbubbles can generate shear stress on the tissue and increase hydraulic conductivity of the interstitial space, leading to increased drug diffusion [26]. Thus, the diffusion of drugs in the perineural and perivascular spaces may be enhanced by the FUS technique. Meanwhile, it has been proposed that the most likely convective mechanism for the widespread distribution of IN administered drugs is the convective flow induced by the expansion and contraction of the perivascular spaces with the cardiac cycle, named “perivascular pump” [4], [37]. Microbubble cavitation can cause expansion and contraction of the perivascular spaces, similar to the expansion and contraction induced by the cardiac cycle but at a much higher frequency [27]. The displacements of the perivascular spaces may induce bulk flow, leading to enhanced convective transport within cerebral perivascular spaces at the FUS targeted region. Furthermore, drugs that enter the systemic circulation through the IN route can cross the BBB at the FUS targeted regions. Therefore, the combination effects of FUS-enhanced local drug diffusion, convective transport, and BBB permeability may contribute to FUS-enhanced IN drug delivery. Ongoing efforts include unveiling the mechanisms by which FUS technique enhances the drug delivery after IN administration.

Whole brain histological examination showed minor microhemorrhages within the FUS targeted region. The damage induced by the FUS technique for FUS+IN and FUS+IV groups were similar (Fig. 4). Figure 5 confirmed that no difference was found in microbubble activities between these two groups. We note that the standard deviations of stable and inertial cavitation doses for FUS+IN group were higher than those for the FUS+IV group. The mice in the FUS+IV group were treated within one day using the same batch of microbubbles; while the mice in the FUS+IN group were treated on separate days using different batches of microbubbles, which could explain the higher standard deviations in microbubble activities observed in the FUS+IN group compared with the FUS+IV group. Nevertheless, these findings suggest that the likelihood of tissue damage by FUS was only correlated with the applied acoustic exposure, not the dextran administration route. The minor damaging effect is consistent with that observed in previous studies [20], [21]. Previous short-term (up to 5-hr survival) and long-term (up to 4-wk survival) safety assessment of the FUS technique demonstrated that the damaging effect, characterized by the presence of a small number of extravasated blood cells within the sonicated region, were found to be transient as no evidence for continuous damage was seen [20], [21]. Behavioral tests performed in non-human primates after repeated FUS sonication in combination with microbubbles showed that FUS did not cause any functional damage even in the presence of tiny clusters of extravasated blood cells [38]. Furthermore, the minimal microhemorrhage could be eliminated by decreasing the FUS exposure [39]. Future studies will optimize the FUS+IN treatment parameters for efficient drug delivery without any tissue damage and explore its potential as a new drug delivery platform in treating CNS diseases.

## Conclusions

IN administration is a promising approach for brain drug delivery. The present study demonstrated for the first time that FUS can enhance IN drug delivery efficiency at the targeted brain location. Despite the fact that the IN route has limited drug absorption across the nasal mucosa, the FUS+IN technique achieved similar drug delivery efficiency within the targeted region compared with the conventional FUS+IV approach. Future studies will explore its potential as a drug delivery platform in treating CNS diseases.
